# A new association of cutaneous leishmaniasis with guselkumab in a patient with chronic psoriasis

**DOI:** 10.1093/skinhd/vzag080

**Published:** 2026-06-12

**Authors:** Yi-Hsuan Chiang, Sarah Winter, Manjunatha Kalavala

**Affiliations:** Dermatology Department, University Hospital of Wales, Cardiff, UK; Dermatology Department, University Hospital of Wales, Cardiff, UK; Dermatology Department, University Hospital of Wales, Cardiff, UK

## Abstract

A 68-year-old White man presented with a 3-month history of two nontender, pruritic, erythematous plaques on his left arm. He had a medical history of chronic plaque psoriasis that has been stable on guselkumab treatment, an anti-interleukin-23 monoclonal antibody, for 5 years. The lesions persisted, despite topical treatments. His medical history also included previous cutaneous squamous cell carcinoma, recurrent penile Bowen disease and psoriasis that responded inadequately to ­multiple biologics before the initiation of guselkumab. A biopsy of a persistent lesion was carried out, which confirmed the diagnosis of cutaneous leishmaniasis. Histopathology demonstrated extensive alignment of amastigotes along the peripheries of histocytes, a pathognomonic sign known as the ‘marquee sign’. Physical examinations and serology were unremarkable. Polymerase chain reaction of the tissue biopsy identified possible involvement of *Leishmania donovani* and *Leishmania mexicana*. Unremarkable computed tomography of the thorax, abdomen and pelvis, and a negative rapid antigen K39 test eliminated the likelihood of visceral involvement. Discussion at a leishmaniasis multidisciplinary team meeting resulted in a final diagnosis of *L. donovani* cutaneous leishmaniasis. The patient recovered well after completing a 4-week course of oral miltefosine. Cases of leishmaniasis associated with the use of biologics associated have so far arisen mainly from tumour necrosis factor-α inhibitors. To our knowledge, this is the first documented case of cutaneous leishmaniasis in a patient treated with guselkumab. Our case emphasizes the importance of considering potential parasitic infections when faced with persistent lesions in patients who are immunosuppressed, especially those receiving biologic therapy.

## Teaser

A 68-year-old White man with a background of chronic psoriasis managed well on guselkumab (an interleukin-23 inhibitor) presented with new plaque-like lesions that did not resolve with topical treatment. A diagnosis of cutaneous leishmaniasis was made after extensive investigations, and the patient later recovered well with a course of oral miltefosine. Untreated leishmaniasis can disseminate beyond the submucosa and may result in airway obstruction or multiorgan failure. We are the first, to our knowledge, to report the association of cutaneous leishmaniasis with guselkumab.

What is already known about this topic?

- Biologic treatments can cause immunosuppression in patients.
- There have been reports of leishmaniasis associated with tumour necrosis factor-α inhibitors and interleukin (IL)-12/23 inhibitors, but there have been no cases associated with IL-23 inhibitors specifically.

What does this study add?

- This is the first documented case of cutaneous leishmaniasis associated with guselkumab, an IL-23 inhibitor.
- We highlight the importance of considering potential parasitic infections in patients with persistent psoriatic lesions while receiving intensive treatments.

Leishmaniasis is a parasitic infection driven by the protozoan *Leishmania* species, which are transmitted through vectors such as female sandflies of the *Phlebotomus* and *Lutzomyia* species. Globally reported cases of leishmaniasis are around 700 000 to 1 million every year, with an estimated 70 000 deaths each year.^1,2^ The severity of leishmaniasis varies greatly depending on the level of dissemination because the risk of fatality increases as the parasite invades further, from a localized cutaneous lesion to ­systemic infection with multiple organ involvement. Known risk factors include travelling to or living in endemic regions, poor socioeconomic status and immunosuppression.^3^

Currently, there are no known indigenous cases in the UK, and no imported cases have been reported in the UK since 2020.^1^ Clinicians may not often include leishmaniasis or other parasitic infections as part of the differential diagnoses due to its rarity outside endemic regions. As a result, missed or delayed diagnosis could lead to poor prognostic outcomes. This case highlights the importance of obtaining a detailed travel history, and of considering potential parasitic infections, even in nonendemic settings.

## Case report

**Figure 1:**
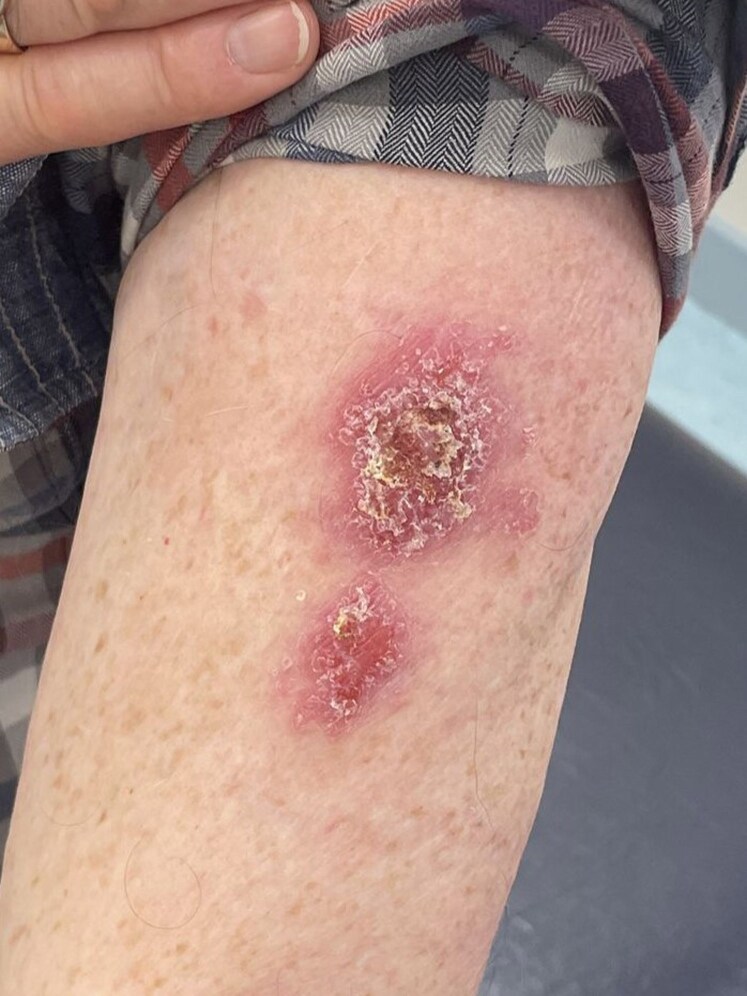
Lesions on the patient’s left upper arm, pretreatment.

A 68-year-old White man presented to our clinic with a 3-month history of two new erythematous, pruritic and nontender plaques located on his left upper arm (Figure 1). He had a background of chronic psoriasis that had been stable on guselkumab [an interleukin (IL)-23 inhibitor] for 5 years. The lesions did not respond to topical therapy. The patient was otherwise well with no other symptoms. Previous biologic treatments for plaque psoriasis included infliximab, etanercept, adalimumab, ustekinumab and secukinumab. These were all discontinued due to inadequate response. The patient also had an extensive and recent travel history to Brazil, Spain, Cape Verde and the Caribbean. His other past medical history included a high-risk squamous cell carcinoma (SCC) of the left lower leg (November 2016) and recurrent penile Bowen disease (April 2006).

**Figure 2:**
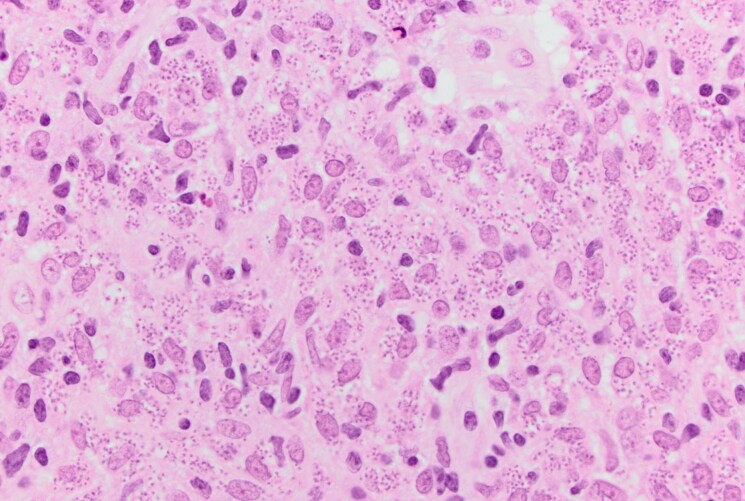
Skin biopsy: amastigotes surrounding the periphery of histiocytes, a classic ‘marquee sign’. Haematoxylin and eosin, ×60 magnification.

Given his history of SCC, a skin biopsy was performed. Histology demonstrated the classic ‘marquee sign’ for leishmaniasis. This is a pathognomonic sign where the peripheries of the histocytes are surrounded by *Leishmania* amastigotes (Figure 2). Physical examination was otherwise unremarkable. Polymerase chain reaction of biopsy tissue suggested that the causative species was *Leishmania mexicana* or *Leishmania donovani*. A rapid antigen k39 test was negative, and unremarkable computed tomography of the thorax, abdomen and pelvis ruled out the likelihood of any visceral involvement.

**Figure 3:**
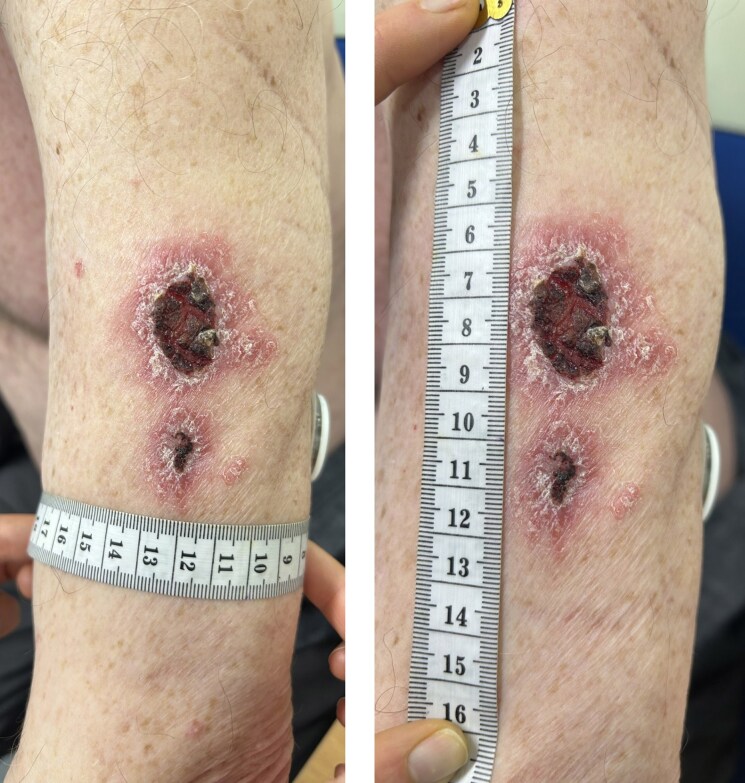
Crusting of lesions after 3 weeks of treatment with miltefosine.

**Figure 4:**
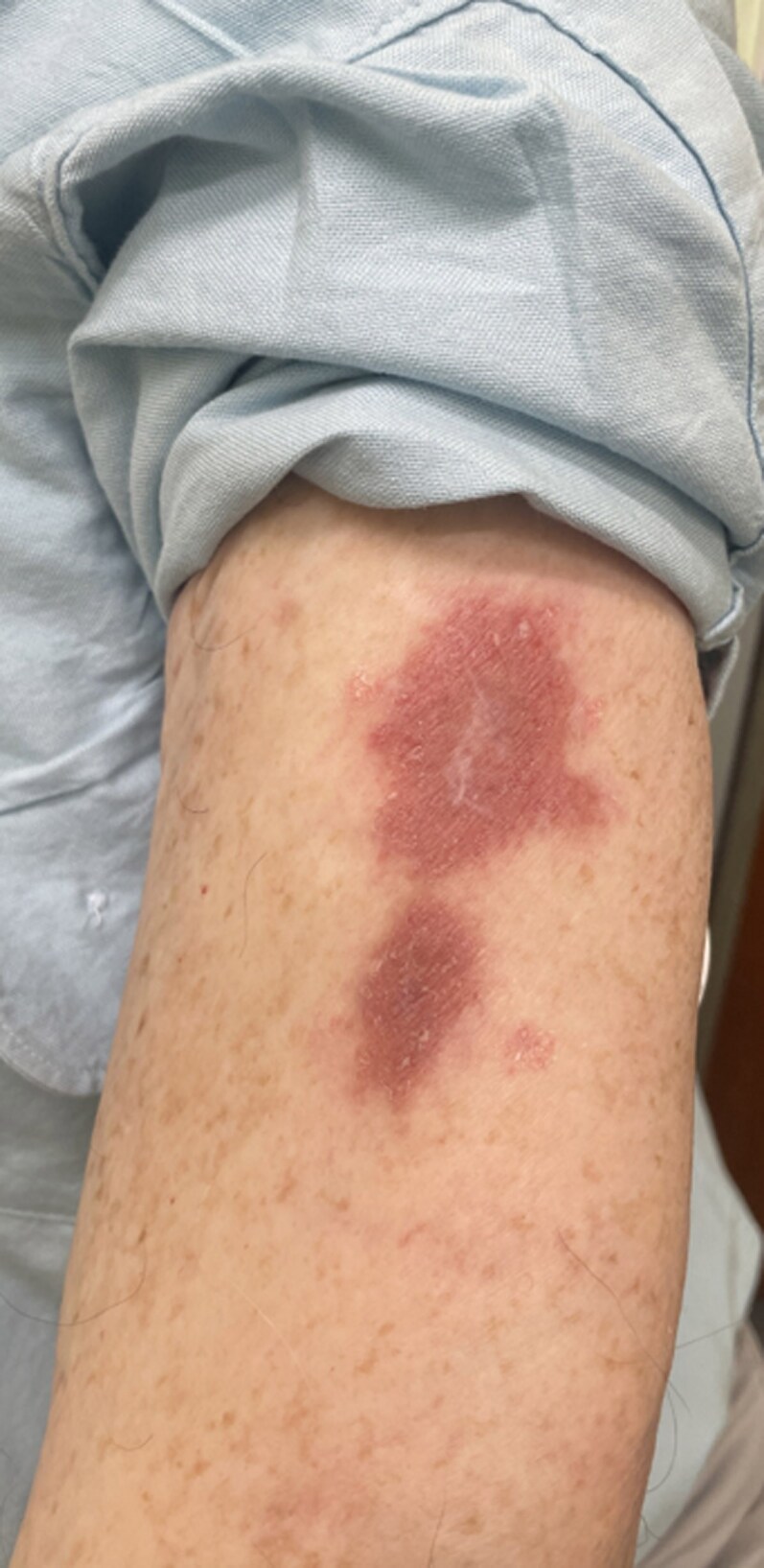
Six weeks post-treatment: only hyperpigmentation remains.

Under joint care with the Infectious Disease team, oral miltefosine was initiated and taken for 4 weeks, resulting in an excellent treatment response on follow-up (Figures 3, 4). This case was later discussed at a leishmaniasis multidisciplinary team meeting, where it was concluded that the diagnosis was most likely cutaneous leishmaniasis due to *L. donovani*, and that the patient would benefit from continuous monitoring for any mucosal involvement.

## Discussion

Leishmaniasis is generally found in tropical regions, and its prognosis differs according to the geographical origin. When contracted in areas primarily including Asia, Africa, the Mediterranean islands, the Middle East and southern Europe, it is classified as ‘Old World’ leishmaniasis, and is generally self-limiting and carries a more favourable prognosis. In comparison, ‘New World’ leishmaniasis (contracted in areas including Central, North and South America) is associated with adverse sequelae.^4,5^

Although cutaneous leishmaniasis is not lethal, its long-term effects cannot be underestimated. The lethality of this parasitic infection increases as the *Leishmania* species disseminates beyond the submucosa. Mucosal involvement poses a risk of airway compromise.^6^ Established guidelines from the USA also endorse continuous monitoring based on otolaryngological symptoms.^7^ In contrast, the deposition of *Leishmania* species in the viscera is associated with the poorest prognosis, with ­*L. donovani* being one of the causative species.^1^ Its presence provokes an excessive and dysregulated immune response, precipitating reactions such as disseminated intravascular coagulation that leads to ischaemic cellular death with progressive fibrosis in the liver and kidney. This cycle can also be aggravated further by superadded bacterial infections, eventually advancing to multiorgan failure and death.^8^

Currently, biologics that have been associated with leishmaniasis are infliximab and adalimumab (tumour necrosis factor-α inhibitor) and ustekinumab (IL-12/23 inhibitor). To date, adalimumab is the only biologic for which an association with leishmaniasis has been reported in the UK.^9–11^

To our knowledge, we are the first to report an association of cutaneous leishmaniasis with guselkumab. The clinical appearance of cutaneous leishmaniasis lesions and those of psoriatic plaques, or even SCC, can be difficult to distinguish on examination. Delayed recognition could carry a high risk of mortality. We highlight the need for clinicians to maintain a high index of suspicion and consider other potential differentials, including parasitic infections, when presented with persistent lesions that may mimic psoriatic or malignant lesions. This is because prompt clinical diagnosis is crucial to ensure that appropriate investigation and treatment are delivered in a timely manner to avoid severe morbidity or mortality.
